# The History and Capabilities of Herbal Simples

**Published:** 1891-10-03

**Authors:** W. T. Fernie


### The History and Capabilities of Herbal Simples.

By W. T. Fernie, M.D.

*(Continued from page 232, Vol. X.)*

### XXXVII.—FERNS (*continued*).

The Royal Fern—*Osmunda regalis*—grows abundantly in many parts of England, and is the stateliest of ferns in its favourite watery haunts. It always needs a soil of bog-earth, and is called, incorrectly, the Flowering Fern, from its handsome spikes of fructification. One of its old English titles is Osmund the Waterman; and the white centre of its root has been called the Heart of Osmund. This middle part, boiled in some kind of liquor, was thought good for persons wounded, drybeaten, and bruised, or that have fallen from some high place. The name Osmund has been supposed to be derived from *os*, the mouth, or *os*, bone, and *mundare*, to cleanse; but others refer it to the Saint Osmund wading a river whilst bearing the Christ on his shoulders. They say that the word Osmund means God's protection, and was first given to the Saxon deity Thor. Anyhow, the Royal Fern has long been associated with the saints and the gods. In the Tyrol it is termed the Blooming Fern, and is placed over the door for good luck. The root, or rhizome, has a mucilaginous, slightly bitter taste. The tender sprigs of the plant at their first comingare "good to be put into balmes, oyles, and healing plasters." A conserve of these tender buds, said Dr. Short, of Sheffield, 1746, is a specific in the rickets; and the root stamped in water or gin till the liquor becometh a stiff mucilage has cured many most deplorable pains of the back that have confined the distracted sufferers close to bed for several weeks. The mucilage was to be rubbed on the vertebræ of the back every night and morning for five or six days together. Also for rickets take of the powdered root, with the whitest sugar, and sprinkle some of this on the child's pap, and on all his liquid foods. It maketh a noble remedy, said Dr. Bowles, without any other medicine. On the whole, the actual curative virtues of this fern are most probably due to the salts of lime, potash, and the like, which it derives in solution from the bog soil, and from the water in which it grows. On July 25th it is specially dedicated to St. Christopher, its patron saint.

The Hart's Tongue, or Hind's Tongue, is a fern of common English growth in shady copses on moist banks, it being the *Lingua cervina* of the apothecaries, and its name expressing the shape of its frond. The older physicians esteemed this as a very valuable medicine, and Galen gave it for diarrhœa or dysentery. It grows often on shady rocks or at the mouth of a well. By reason of its tannin it restrains hemorrhages, being commended, says Gerarde, against the bloody flux. Common people employ an ointment made from its leaves for burns and scalds. When fully ripe, its seeds are thrown to a considerable distance by the elastic ring of each vessel which contains them, beneath the leaves. Of the five great capillary herbs formerly held in high repute, this and the common Maidenhair have alone been retained in the Edinburgh Pharmacopœia. Dr. Tuthill Massy advises the drinking in Bright's disease of as much as three half-pints daily of an infusion of this plant, whilst always taking care to gather the young shoots. Also in combination with the American Golden Seal—*Hydrastis Canadensis*—the Hart's Tongue has certainly served in several authenticated cases to arrest the progress of that formidable disease, diabetes mellitus. Its distilled water is commended to be taken against palpitations of of the heart and to stay the hiccough; also to help the falling of the palate or stop bleeding of the gums, if the mouth be gargled therewith.

The common Polypody fern grows abundantly in this country on old walls and stumps of trees and in shady places. In Hampshire it is called adder's tongue, as derived really from the word "attor," poison. In Germany it is said to spring from the Virgin's milk, and is named Marie bregue. The fresh root has been used with success in maniacal melancholia. By the ancients it was employed as a purgative. Six drams by weight of this fresh root should be infused for two hours in a pint of boiling water and given in two doses. The same is the oak fern of the herbalists—not that of modern botanists—it being held that such fern plants as grew upon the roots of an oak tree were of special medicinal powers—"*quod nascit super radices quercus est efficacius.*" Dioscorides said the root of polypody is very good for chaps between the fingers. "It serveth," writes Gerarde, "to make the belly soluble, being boiled in the broth of an old cock, with beets or mallows or other like things that move to the stool by their slipperiness." Parkinson says, "A dram, or two, if need be, of the powdered dry roots, taken fasting, in a cupful of honeyed water, worketh gently as a purge, being a safe medicine, fit for all persons and seasons, which daily experience confirmeth. Applied also to the nose it cureth the disease called polypus, which by time and sufferance stoppeth the nostrils." The true oak fern of botanists—*Polypodium dryopteris*—grows chiefly in mountainous districts among the mossy roots of old oak trees, and sometimes in marshy places. If its root is bruised and applied to the skin of any hairy part whilst the person is sweating this will cause the hair to come away. The true Maidenhair fern (*Adiantum capillus veneris*) of exquisite foliage, and of a dark crimson colour, is a stranger in England, except in the West country. From its fine hair-like stems, and perhaps from its attributed virtues in toilet use, this fern has acquired the name of "Our Lady's Hair" and "Maria's Fern." "The true maidenhair," says Gerarde, "maketh the hair of the head and the beard to grow that is fallen and pulled off." From this beautiful fern a famous elegant syrup is made in France called capillaire, and is given as a favourite remedy in pulmonary catarrh. It is flavoured with orange flowers, and acts as a demulcent with slightly stimulating effects. One part of the drug is gently boiled with ten parts of water, and with nineteen parts of white sugar. In England we have more abundantly the common maidenhair—*Asplenium Trichomanes*—of which the leaves are likewise useful in pulmonary disorders, being sweet and mucilaginous and expectorant. But Sir John Hill ordered that (as we cannot get the true plant fresh) the fine syrup made in France from the maidenhair in perfection with pure Narbonne honey is by no means a trifle; because barley-water sweetened with this is one of the very best remedies for a violent cold. The common maidenhair, which grows on old walls, is a laxative; and idiots are said to have recovered their reason by occasionally employing it medicinally. The true plant, "which strengthens and embellishes the hair," occurs but rarely with us, on damp rocks, and walls near the sea. Theophrastus said it is "*ad defluvium capillorum utile.*"

This true maidenhair is called Adiantum, from the Greek, *quod denso imbre cadente, destillans foliis tenuis non insidet humor*—the leaves are not wetted even by a heavily falling shower of rain. "In vain," saith Pliny, "do you plunge the adiantum in water; it always remains dry." A tea brewed from this plant answers the same purpose as the syrup for tedious coughs. Again, the wall rue(*Ruta muraria*) is a white maidenhair fern, and is called by some *Salvia vitæ*. It is a small herb, somewhat nearly of the colour of garden rue, and is likewise good for them that have a cough, or are short-winded, or be troubled with stitches in the sides. It stayeth the falling or shedding of the hair, and causeth them to grow thick, fair, and well coloured. This plant is held by those of judgment and experience to be as effectual a capillary herb as any whatever. Also it helpeth ruptures in children. Matthiolus hath known of divers holpen therein by taking the powder of the herb in drink for forty days together.

The finger fern—*Asplenium ceterach* (an Arabian term)—grows on old walls and in the clefts of moist rocks. A decoction made with the whole plant in boiling water acts as a useful stimulant to the liver if given when that organ is sluggish. This plant has been long known by the name of spleenwort, or miltwaste, because thought to remove disorders of the spleen.

"The finger fern, which being given to swine
It makes their milt to melt away in fine."

Probably the shape of the leaf, by resembling the form of the spleen, has chiefly conferred this reputed virtue. "No herbe maie be compared therewith," says one of the oldest herbals, "for his singular vertue to help the sicknesse, or grief of the splene." Pliny ordered it should not be given to women because it bringeth barrenness. There grows in Tartary a singular polypody fern, of which the hairy root is easily made to resemble in form a small sheep. It rises above the ground, with excrescences simulating a head and a tail, whilst having four leg-like fronds. Fabulous stories are related about this remarkable fern root; and in China its hairy down is so highly valued as a styptic for fresh-bleeding cuts and wounds, that few families will be without it. Dr. Darwin, in his "Loves of the Plants," says about this curious natural production:

"It eyes with mute appeal a distant dam,
And seems to bleat—*a Vegetable Lamb.*"

